# Chemotherapy-induced functional brain abnormality in colorectal cancer patients: a resting‐state functional magnetic resonance imaging study

**DOI:** 10.3389/fonc.2022.900855

**Published:** 2022-07-18

**Authors:** Siwen Liu, Yesong Guo, Jie Ni, Na Yin, Chenchen Li, Xuan Pan, Rong Ma, Jianzhong Wu, Shengwei Li, Xiaoyou Li

**Affiliations:** ^1^ Research Center for Clinical Oncology, Jiangsu Cancer Hospital & Jiangsu Institute of Cancer Research & The Affiliated Cancer Hospital of Nanjing Medical University, Nanjing, China; ^2^ Department of Radiotherapy, Jiangsu Cancer Hospital & Jiangsu Institute of Cancer Research & The Affiliated Cancer Hospital of Nanjing Medical University, Nanjing, China; ^3^ Department of Oncology, Jiangsu Cancer Hospital & Jiangsu Institute of Cancer Research & The Affiliated Cancer Hospital of Nanjing Medical University, Nanjing, China; ^4^ Department of Radiology, Jiangsu Cancer Hospital & Jiangsu Institute of Cancer Research & The Affiliated Cancer Hospital of Nanjing Medical University, Nanjing, China; ^5^ Department of Anorectal, Yangzhou Traditional Chinese Medicine Hospital Affiliated to Nanjing University of Chinese Medicine, Yangzhou, China

**Keywords:** chemotherapy, cognitive impairment, resting-state functional magnetic resonance imaging, fractional amplitude of low-frequency fluctuation, colorectal cancer

## Abstract

**Introduction:**

Chemotherapy-induced cognitive impairment (i.e., “chemobrain”) is a common neurotoxic side-effect experienced by many cancer survivors who undergone chemotherapy. However, the central mechanism underlying chemotherapy-related cognitive impairment is still unclear. The purpose of this study was to investigate the changes of intrinsic brain activity and their associations with cognitive impairment in colorectal cancer (CRC) patients after chemotherapy.

**Methods:**

Resting‐state functional magnetic resonance imaging data of 29 CRC patients following chemotherapy and 29 matched healthy controls (HCs) were collected in this study, as well as cognitive test data including Mini Mental State Exam (MMSE), Montreal Cognitive Assessment (MoCA) and Functional Assessment of Cancer Therapy-Cognitive Function (FACT-Cog). The measure of fractional amplitude of low-frequency fluctuation (fALFF) was calculated and compared between groups. The correlations between the fALFF of impaired brain region and cognitive performance were also analyzed.

**Results:**

Compared with HCs, CRC patients following chemotherapy showed decreased fALFF values in the left anterior cingulate gyrus (ACG) and middle frontal gyrus, as well as increased fALFF values in the left superior frontal gyrus (orbital part) and middle occipital gyrus. Moreover, positive associations were identified between fALFF values of the left ACG and the total scores of MMSE, MoCA and FACT-Cog in the patient group.

**Conclusion:**

These findings indicated that CRC patients after chemotherapy had decreased intrinsic brain activity in the left ACG, which might be vulnerable to the neurotoxic side-effect of chemotherapeutic drugs and related to chemotherapy-induced cognitive impairment.

## Introduction

Colorectal cancer (CRC) is one of the malignant diseases and has high incidence of mortality (1). Approximately 25% of patients newly diagnosed with CRC displayed metastasis, and roughly 50% of those eventually developed metastasis during their lifetime struggling with cancer (2). Advances in early detection, diagnosis and treatment have led to significant improvements in the five-year survival rates of patients with CRC (3). The available treatment options for CRC include surgery, radiotherapy, immunotherapy, targeted therapy (either alone or in combination) and chemotherapy (4). A high number of cancer patients who received chemotherapy had self-reported psychological and cognitive impairment, which had a negative impact on the quality of life (QoL) for cancer patients (5). Chemotherapy related cognitive impairment is called “chemo-brain”, which is a complex treatment-related side effect experienced by cancer survivors during chemotherapy. In addition, cancer related inflammation, anemia, malnutrition and infection, as well as anxiety/depression, negative thoughts, rumination/worry, sleep disorders caused by cancer, might also lead to cognitive impairment (6). Recommended chemotherapeutic drugs for CRC include 5-fluorouracil (5-FU) with leucovorin and irinotecan or oxaliplatin, alone or combined with bevacizumab (7). Although these drugs are effective at improving overall survival (OS) of CRC patients, severe side-effects, such as chemotherapy-related neurotoxicity including toxicity in the central nervous system (CNS), were often found in patients after receiving chemotherapy. The chemotherapy-induced central neurotoxicity were considered to be associated with the endogenous mechanisms, which were named the target and the blood-brain barrier transporter hypotheses (8). Therefore, assessing chemotherapy-related negative impacts on neurocognitive function is crucial for the development of preventive measures and rehabilitation programs after chemotherapy. Additionally, the central pathophysiological mechanism of cognitive impairment associated with chemotherapy is complicated and still unclear.

Cognitive impairment was found in 13%-57% of CRC patients treated with chemotherapy (9), which was similar to the study reporting the prevalence of cognitive impairment ranged from 17% to 75% among patients of various cancer types (10). Another study found that 18-24% of CRC patients had perceived cognitive impairment before treatment based on the Functional Assessment of Cancer Therapy-Cognitive Function (FACT-Cog) while the impaired cognition in those who treated with chemotherapy was aggravated (11). Moreover, the impaired cognition was related to anxiety, depression, fatigue and poorer QOL, which suggested that these negative concomitant symptoms might be associated with cancer-related distress or negative appraisal and decreased tolerance or resilience to manage cancer-related pain and chemotherapy-related side-effects (11–13). A meta-analysis study also found that older CRC patients were more vulnerable to cognitive impairment after chemotherapy, and preventive measures and rehabilitation programs could reduce the risk of developing chemotherapy-induced cognitive impairment (14). A prospective, longitudinal, controlled study showed that 36%-52% CRC patients had cognitive impairment including working memory, attention, verbal memory and processing speed (15). Patients treated with chemotherapy regimens of capecitabine with/without oxaliplatin had high risk of developing cognitive impairment while FOLFOX (folate, 5-FU, oxaliplatin) and oxaliplatin was negatively related to the cognitive impairment (16). In addition, FOLFIRI regimen (folate, 5-FU, irinotecan) caused to increased risk of cognitive impairment in older CRC patients (16). Patients with CRC were also found to have decreased executive function after 12 months of chemotherapy (5-FU with or without oxaliplatin) (17).

Resting-state functional magnetic resonance (rs-fMRI) is a non-invasive imaging method with high spatial resolution, which can be used to detect functional activity during rest by measuring intrinsic blood oxygen level-dependent (BOLD) low-frequency signal fluctuations (18). Therefore, the method of rs-fMRI has been extensively used to explore the central pathophysiological mechanism of cognitive impairment and provide neurophysiological evidence for functional changes in the brain of patients with cognitive impairment (19, 20). Among commonly used measures, the amplitude of low-frequency fluctuation (ALFF) and fractional ALFF (fALFF) with higher test-retest reliability have been applied to measure regional spontaneous brain activity (21, 22). ALFF is calculated as the square root of the power spectrum in low-frequency range, which measures the amplitude of spontaneous brain activity during rest (22). However, the measure of ALFF is sensitive to the physiological noise (23). Therefore, to overcome this limitation, the measure of fALFF measuring the ratio of the low-frequency power spectrum to that of the entire frequency range, is proposed as an improved ALFF indicator, which has been considered as a more robust measure for regional spontaneous brain activity (21). Based on the measure of ALFF, patients with breast cancer receiving one month chemotherapy had increased brain activity in the bilateral precuneus and middle temporal gyrus, left upper and inferior temporal gyrus, which mainly located in the default mode network (DMN) (24). Patients with gastric cancer had decreased ALFF in the bilateral fontal and temporal areas after chemotherapy (capecitabine+oxaliplatin) and decreased fALFF was found in the anterior cingulate gyrus (ACG) and bilateral orbitofrontal gyrus when compared with healthy controls (HCs) (25). Decreased fALFF was identified in the left precuneus of breast cancer patients received a chemotherapy regimen of docetaxel and cyclophosphamide (26). Lung cancer patients before chemotherapy demonstrated decreased ALFF in the right posterior cingulate cortex when compared with patients after chemotherapy including pemetrexed, gemcitabine, cisplatin, nedaplatin, docetaxel and etoposide (27). Therefore, we speculated that fALFF might be a promising imaging indicator for exploring the central pathological mechanism of cognitive impairment in CRC patients after chemotherapy.

To the best of our knowledge, this is the first study to explore the central pathological mechanism involving in the cognitive impairment of CRC patients who had undergone chemotherapy. Based on previous neuroimaging findings, we hypothesized that CRC patients with chemotherapy-related cognitive impairment might have altered functional activity in some brain regions. Therefore, the method of rs-fMRI and the measure of fALFF were applied to explore the possible central mechanism underlying cognitive alternations of CRC patients after chemotherapy in this study.

## Materials and methods

### Participants

In total, 29 CRC patients who had undergone chemotherapy were recruited from the Department of Oncology, Jiangsu Cancer Hospital & Jiangsu Institute of Cancer Research & The Affiliated Cancer Hospital of Nanjing Medical University, Nanjing, China. The inclusion criteria of CRC patients were as follows: 1) histological confirmation of CRC adenocarcinoma; 2) received 2 to 3 months of chemotherapy with standard chemotherapeutic agents; 3) right-handed;4)aged from 40 to 70 years; 5) received at least 9 years of education. The exclusion criteria for patients were as follows: 1) terminal stage of CRC (survival period <1 year); 2) history of diagnosis and treatment for any other cancer; 3) presence of brain metastasis; 4) history of radiation therapy before the enrollment; 5) presence of any major medical illness; 6) psychiatric or neurological disorders; 7) history of substance abuse; 8) any contraindication of MRI scans. According the inclusion and exclusion criteria of patients, 29 age, sex, handness and education level-matched HCs with no history of any cancer were enrolled through the online advertisement. The sample size was estimated as follows: δ=(μ_D_-0)/, where δ was the effect size, μ_D_ was the difference in means between groups, 0 was the difference in means under the null hypothesis, and δ was the variability in the difference in means (28). In fMRI, μ_D_ and δ were typically normalized as percent signal change (i.e. 100×(*E*-*C*)/*C*, where *E*, patients and *C*, HCs) (28).

This study was approved by the Research Ethics Committee of Jiangsu Cancer Hospital & Jiangsu Institute of Cancer Research & The Affiliated Cancer Hospital of Nanjing Medical University. All subjects signed informed consent before their participation in the study.

### Cognitive function assessment

The cognitive function of all participants was measured using the self-report questionnaires including Mini Mental State Exam (MMSE) (attention, memory, language, and visuospatial abilities, as well as orientation to person, place and time) (29), Montreal Cognitive Assessment (MoCA) (attention and concentration, executive function, memory, language, visual structure skills, abstract thinking, calculation and orientation) (30) and Functional Assessment of Cancer Therapy-Cognitive Function (FACT-Cog) (patients’ perceived cognitive impairments, perceived cognitive abilities, noticeability or comments from others, impact of cognitive changes on quality of life) (31).

### MRI data acquisition and preprocessing

**Figure 1:**
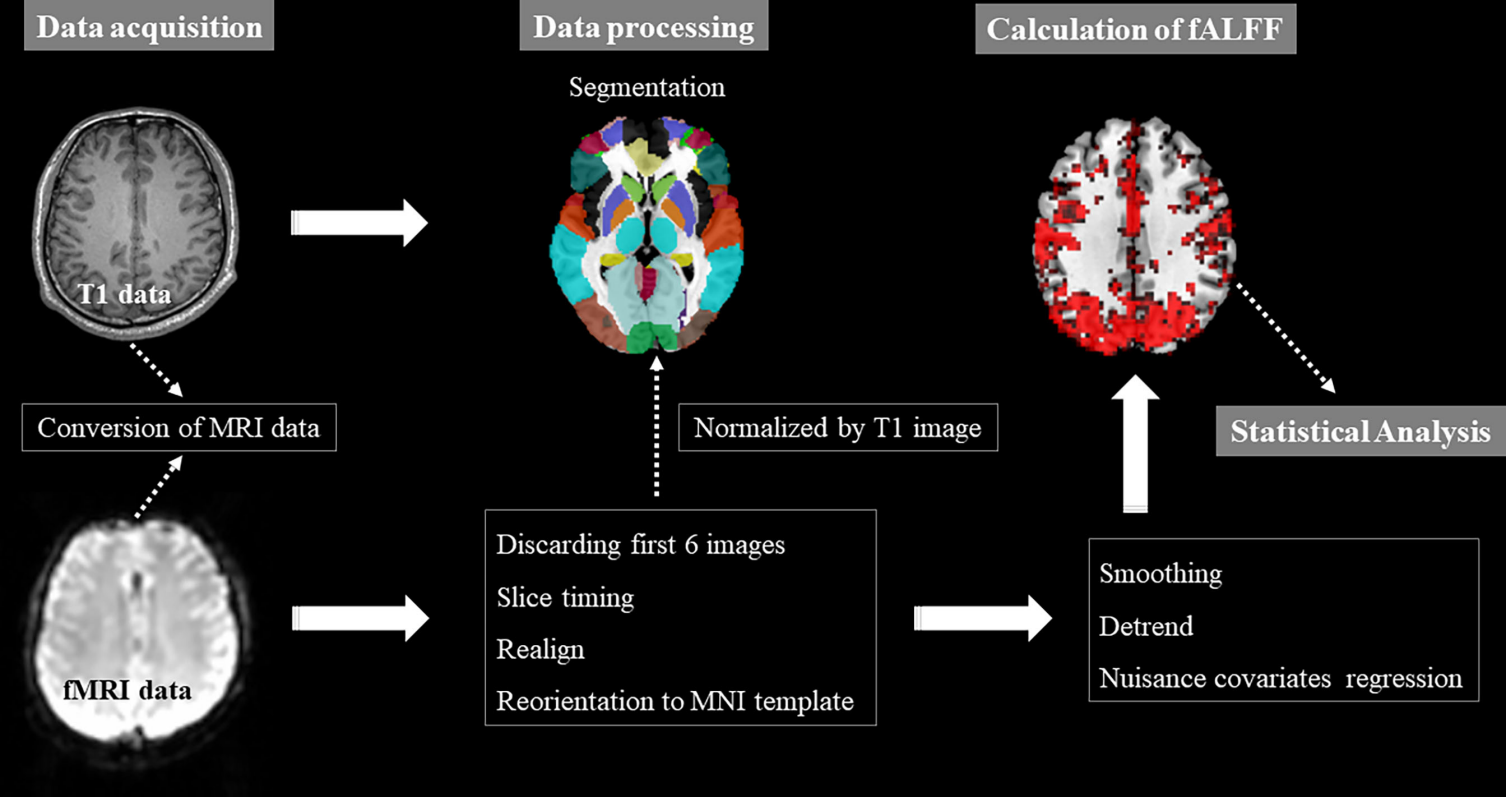
Pipeline of MRI data acquisition and processing, and fALFF calculation. fALFF, fractional amplitude of low-frequency fluctuation; fMRI, functional magnetic resonance; MNI, Montreal Neurological Institute; x, y and z: the coordinates of peak voxel of each cluster in the MNI space.

MRI data were acquired following the complete treatment of chemotherapy (2 to 3 months) with a 3.0 T Philips Aachieva scanner (**Figure 1**). The details about parameters of MRI acquisition were described in our previous study (**Figure 1**) (32). The MRI data preprocessing was conducted using the Data Processing Assistant for rs-fMRI advanced edition (DPARSF) in MATLAB software (33). The whole preprocessing procedures were as follows: 1) conversion of MRI data; 2) discarding first 6 images; 3) slice timing; 4) realign; 5) reorientation of images to the Montreal Neurological Institute (MNI) template; 6) segmentation; 7) normalized by T1 image (3×3×3mm^3^ resolution); 8) smoothing with a 4 mm Gaussian kernel; 9) detrend; 10) nuisance covariates regression including signals from white matter and cerebrospinal fluid and Friston-24 head motion parameters. Participants with head motion >2mm or a spin of >2° in any direction were excluded in this study.

### Calculation of fALFF

The measure of fALFF was calculated by the software DPARSF (**Figure 1**). Firstly, the time course of each voxel in the brain was converted to the frequency domain without band-pass filter by the Fast Fourier transform (FFT) and then the power spectrum was obtained. Secondly, the square root at each frequency of the power spectrum was calculated, and the average square root of the power spectrum of each voxel across 0.01-0.08 Hz was calculated as the ALFF value of voxel. The ratio of the power spectrum in the low-frequency band (0.01-0.08 Hz) to that in the whole frequency range was defined as fALFF value. Finally, to reduce the nonspecific signals, fALFF maps were obtained through fALFF value of each voxel divided by the global mean fALFF value. ALFF is a measure for exploring spontaneous brain activity, however, it is often influenced by physiological noise. Therefore, to overcome the limitations of ALFF, fALFF is proposed to selectively suppress artifacts from non-specific brain areas.

### Statistical analysis

With the software of SPSS (version 23.0; IBM, Armonk, NY, USA), the two-sample *t*-test was used to analyze the differences of age, educational level, scores of MMSE and MoCA between groups while the Chi-squared test was performed to estimate the differences of gender distribution. Statistical significance was defined as *P*<0.05.

In addition, to identify the differences of fALFF between groups, the two-sample *t*-test was conducted by the software of Resting-State fMRI Data Analysis (REST) Toolkit (34). The significance threshold was set at *P*<0.05 (voxel-level was set at *P*<0.001 and cluster-level was set at *P*<0.05; two tailed; the corresponding minimum cluster size was 21 voxels) for multiple comparisons using Gaussian Random Field (GRF) theory.

Finally, *Pearson* correlation analyses were conducted to examine whether the fALFF values of brain regions showing group-differences were correlated with the total scores of cognitive scales. The significance level was set at *P*<0.05.

## Results

### Demographics and clinical characteristics

A total of 29 CRC patients (0 in stage I, 3 in stage II, 20 in stage III and 6 in stage IV) were enrolled in this study. In all patients, 5 patients had lesions in the colon while 23 patients had lesions in the rectum. In addition, 5 patients had no metastasis while other cases had metastasis in the lung (2), liver (14), bone (1), as well as intraperitoneal (6) and pelvic metastasis (1). Two chemotherapy regimens were used in this study including CAPOX (capecitabine plus oxaliplatin) (10 patients) and CAPOX plus bevacizumab (19 patients). The drugs in the chemotherapy regimens were administered in accordance with the National Comprehensive Cancer Network (NCCN) guidelines for CRC (35, 36). Moreover, there were no differences in the age, gender and educational level between groups. However, patients had decreased scores of MMSE, MoCA and FACT-Cog when compared with those of HCs. The demographics and clinical information of two groups were presented in **Table 1**.

**Table 1:** Demographic and clinical characteristics.

Variables	CRC (n=29)	HCs (n=29)	*t*/χ^2^	*P*
**Age (years)**	58.21 ± 8.55	56.97 ± 7.02	0.60	0.55^a^
**Gender (male/female)**	14/15	18/11	1.12	0.29^b^
**Education level (years)**	14.03 ± 1.57	14.62 ± 1.45	-1.48	0.15^a^
**Cognitive function assessment**				
Scores of MMSE	25.62 ± 1.57	26.55 ± 1.21	-2.53	0.014^a^
Scores of MoCA	27.00 ± 0.96	27.55 ± 0.69	-2.51	0.015^a^
Scores of FACT-Cog	98.21 ± 4.46	100.38 ± 3.48	-2.07	0.043^a^
**Location: n (%)**				
Colon	5 (17%)	–	–	–
Rectum	24 (83%)	–	–	–
**Disease stage: n (%)**				
I	0 (0%)	–	–	–
II	3 (10%)	–	–	–
III	20 (70%)	–	–	–
IV	6 (20%)	–	–	–
**Metastasis: n (%)**				
No	5 (17%)	–	–	–
Lung	2 (7%)	–	–	–
Liver	14 (48%)	–	–	–
Bone	1 (3.5%)	–	–	–
Intraperitoneal	6 (21%)	–	–	–
Pelvic	1 (3.5%)	–	–	–
**Chemotherapy regimen: n (%)**				
CAPOX	10 (35%)	–	–	–
CAPOX plus bevacizumab	19 (65%)	–	–	–

CRC, colorectal cancer; HCs, healthy controls; MMSE, Mini Mental State Exam; MoCA, Montreal Cognitive Assessment; FACT-Cog, Functional Assessment of Cancer Therapy-Cognitive Function; CAPOX, capecitabine plus oxaliplatin. P<0.05 was considered to be statistically significant. ^a^, P values were obtained using two sample t-tests. ^b^, P value was obtained using the Pearson chi-square test.

### Differences of fALFF between groups

**Figure 2:**
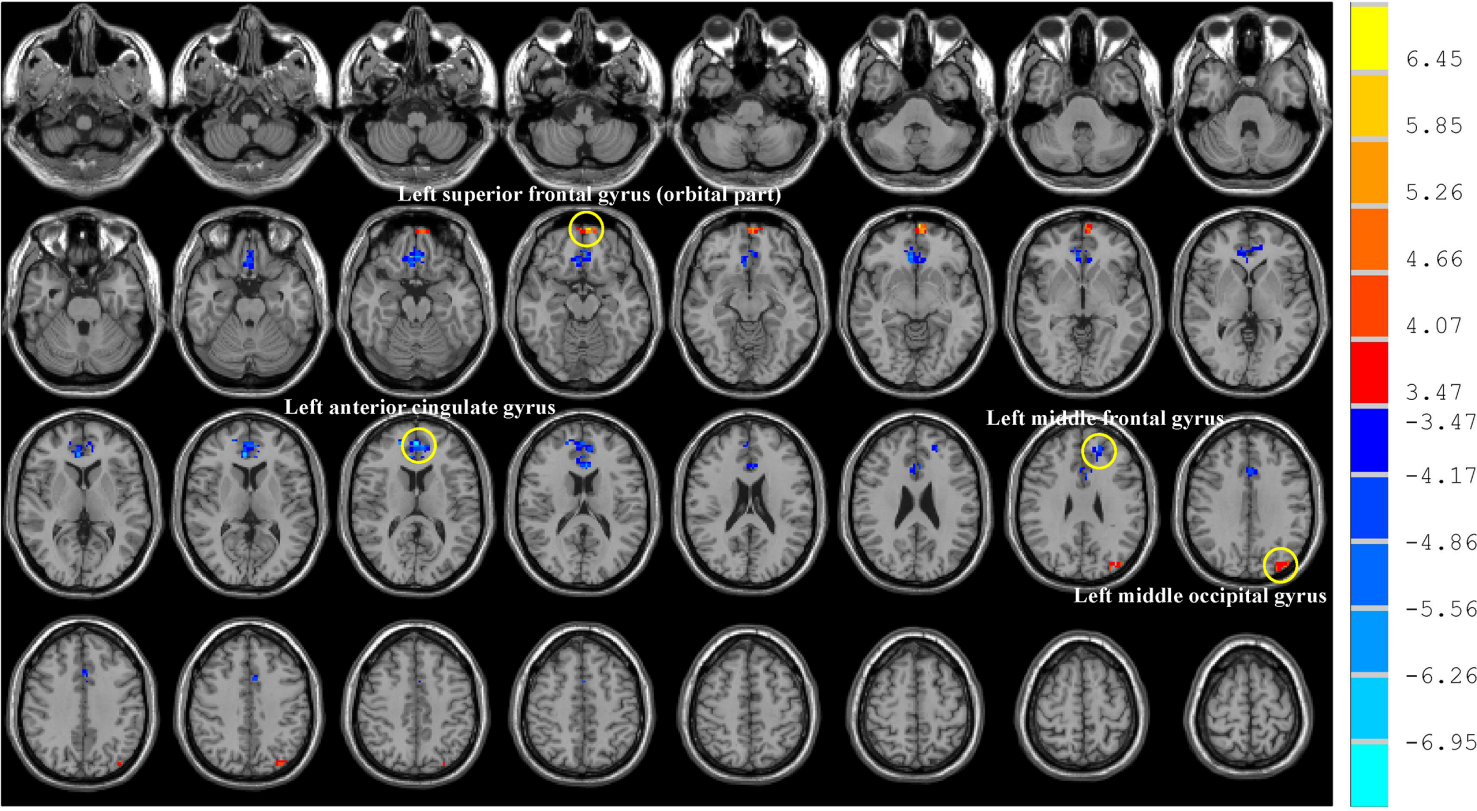
Brain regions showed altered fALFF in CRC patients. Post-treatment CRC patients showed decreased fALFF in the left anterior cingulate gyrus and middle frontal gyrus, while displaying increased fALFF in the left superior frontal gyrus (orbital part) and middle occipital gyrus. CRC: colorectal cancer. fALFF: fractional amplitude of low-frequency fluctuation. The significance threshold was set at *P*<0.05 (voxel-level was set at *P*<0.001 and cluster-level was set at *P*<0.05; two tailed; the corresponding minimum cluster size was 21 voxels) for multiple comparisons using Gaussian Random Field (GRF) theory. Red indicated regions with increased fALFF while blue indicated regions with decreased fALFF.

The fALFF values of patients after chemotherapy showed a significant decrease in the left ACG and middle frontal gyrus, while displaying a significant increase in the left superior frontal gyrus (orbital part) and middle occipital gyrus when compared with those of HCs (**Table 2**; **Figure 2**). In addition, we also compared the differences of fALFF values between patients received different chemotherapy regimen [CAPOX (10 patients) vs CAPOX plus bevacizumab (19 patients)] and details about the participants, methods and results were illustrated in **Supplementary Materials**.

**Table 2:** Brain regions showed altered fALFF in CRC patients.

Brain regions	Peak MNI coordinates			Clusters	Peak T values
	x	y	z		
Left anterior cingulate gyrus	0	51	12	404	-7.65
Left superior frontal gyrus (orbital part)	-6	66	-15	55	7.05
Left middle frontal gyrus	-21	48	27	24	-5.33
Left middle occipital gyrus	-39	-84	33	41	5.08

CRC, colorectal cancer; HCs, healthy controls. fALFF, fractional amplitude of low-frequency fluctuation. MNI, Montreal Neurological Institute; x, y and z, the coordinates of peak voxel of each cluster in the MNI space. The significance threshold was set at P<0.05 (voxel-level was set at P<0.001 and cluster-level was set at P<0.05; two tailed; the corresponding minimum cluster size was 21 voxels) for multiple comparisons using Gaussian Random Field (GRF) theory.

### Correlations between fALFF values and scores of cognitive scales

**Figure 3:**
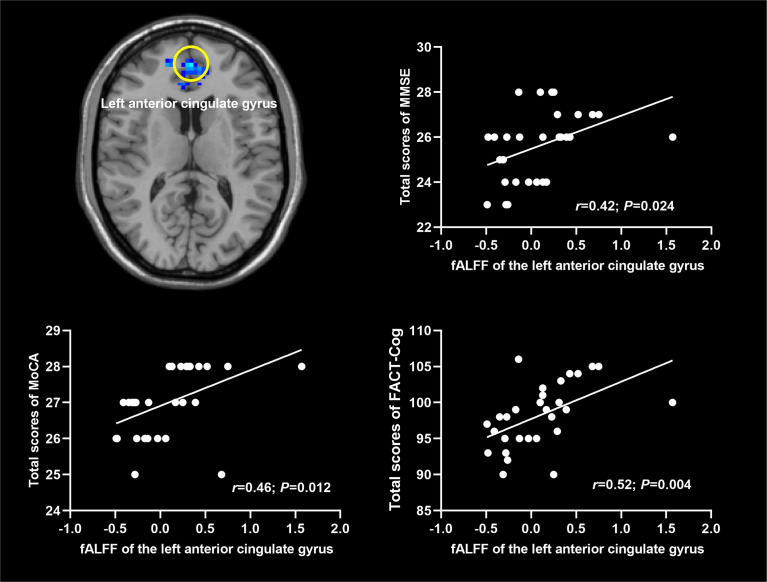
Correlations between fALFF and scores of cognitive scales. fALFF: fractional amplitude of low-frequency fluctuation. MMSE: Mini Mental State Exam; MoCA: Montreal Cognitive Assessment; FACT-Cog: Functional Assessment of Cancer Therapy-Cognitive Function. The significance level was set at *P*<0.05.

As shown in **Figure 3**, fALFF values of the left ACG were positively correlated with the total scores of MMSE (*r*=0.42; *P*=0.024), MoCA (*r*=0.46; *P*=0.012) and FACT-Cog (*r*=0.52; *P*=0.004).

## Discussion

In this study, we utilized rs-fMRI to investigate the changes of spontaneous brain activities in CRC patients following chemotherapy compared to HCs with the measure of fALFF. We found that patients presented with decreased scores of cognitive function assessment, which suggested that chemotherapy might lead cognitive dysfunction. patients also had decreased fALFF in the left ACG and middle frontal gyrus, and increased fALFF in the left superior frontal gyrus (orbital part) and middle occipital gyrus, which suggested different patterns of spontaneous brain activity between groups in these regions. In addition, we found significant positive correlations between fALFF values of the left ACG and scores of cognitive scales including MMSE, MoCA and FACT-Cog.

Chemotherapy has neurotoxicity on both the central and peripheral nervous system, which manifests as a wide range of clinical syndromes including cognitive impairment (37, 38). Increasing evidence demonstrated that chemotherapeutic drugs (cytotoxic drugs) for non-central nervous system cancer might also lead to cognitive impairment related to chemotherapy (39, 40). The relationship between chemotherapy and cognitive performance of cancer patients who undergone chemotherapy had been explored in previous studies (41–43). In this study, CRC patients had decreased scores of MMSE, MoCA and FACT-Cog, which implied that these patients following chemotherapy had impaired cognitive function. This finding was consistent with the result of previous study, which showed that chemotherapy had a negative effect on the cognitive function of patients (56%) with colon cancer, such as verbal memory (44). Patients with other cancer also had subjective cognitive dysfunction after chemotherapy and the most serious effect was found at one month after chemotherapy, which suggested that the impact of chemotherapy might be an acute rather than a chronic side effect on cognitive function (45).

ACG is one of subregions in the prefrontal cortex, which is considered to be involved in a broad range of processes including cognitive control, emotion processing and attention (46–49). Therefore, it is a critical part of the neural network implicated in cognitive control process, such as attentional control, conflict monitoring and error detection, and it is active when cognitive control is required (50–52). As an important brain region implicated in cognitive control, it has been hypothesized that the activation of ACG increases top-down control (53–55). Reduced functional connectivity was identified in ACG of patients with mild cognitive impairment (MCI), which supported the idea that abnormal activity of ACG might be a useful indicator to understand the central pathological mechanism of cognitive impairment, as well as sensitive neuroimaging biomarker for the early detection of MCI (19, 56, 57). In addition, impaired structure of ACG was also found in patients with MCI and it was paralleled with decreased performance of cognitive function, especially interference and conflict monitoring (58). The structural abnormality of ACG was identified as a predictor of cognitive impairment, which suggested that ACG played a key role in the underlying pathogenesis of cognitive impairment (59). In addition, the impaired cognitive function could be improved by mind-body exercise (movement-based mind-body intervention), which could modulate regional brain activities and structure in ACG (60). The cognitive improvement was also found to be associated with increased functional connectivity of ACG in patients with cognitive impairment (61).

In this study, we found that CRC patients had decreased brain activity in left ACG when compared to HCs. Previous study provided evidence that chemotherapeutic drug, cisplatin, could lead to the abnormality of ACG neurons, which might be associated with cognitive deficits of rats following chemotherapy (62). Cisplatin had been shown to effectively block the expression of local field potential (LTP) at amygdala-ACG synapses and affect the physiological expression of theta oscillation activity (62). These findings suggested that cisplatin might contribute to desynchronization in the amygdala-ACG, which were associated with the abnormalities of learning and memory (62). A pattern of dispersion of theta band activity and reduction in correlation values of cisplatin were found when simultaneously recording local field potentials in ACG and basolateral amygdala (BLA), which might be relevant to the failure of introduction of LTP in the BLA- ACG circuit after administration of cisplatin (62). In human, non-CNS cancer patients after chemotherapy exhibited reduced grey matter volume in ACG, which might contribute to cognitive impairment related to chemotherapy (63). In addition, breast cancer patients received 6 months of chemotherapy showed reduction of grey matter volume in ACG (64). Decreased functional connectivity was also found in breast cancer patients after receiving chemotherapy, which suggested that abnormal function in ACG might be implicated in the cognitive impairment related to chemotherapy of breast cancer patients (65). Importantly, decreased functional connectivity was found in ACG of post-chemotherapy patients with lung cancer (chemotherapy regimen: cisplatin based therapy or carboplatin based therapy for at least six months), and positive association was found between decreased functional connectivity in ACG and reduced MoCA scores of patients (66). Moreover, gray matter density of ACG was also found to decrease in lung cancer patients following platinum-based chemotherapy (67). ACG is considered to play an important role in cognitive control (68). Therefore, these findings supported the view that impaired ACG might play a key role in the development of cognitive impairment in cancer patients treated with chemotherapy. However, it was difficult to make causal relationships between functional abnormalities of ACG and cognitive impairment due to the cross-sectional experimental design. Overall, additional studies with larger sample size were necessary to confirm these results, and longitudinal studies were needed to explore the causal relationships between the functional abnormalities of ACG and chemotherapy-induced cognitive impairment in CRC patients, and to better understand the mechanisms underlying “chemo-brain”.

## Conclusion

In summary, we observed chemotherapy-associated cognitive impairment in CRC patients following chemotherapy, which might be associated with decreased fALFF in the left ACG. These findings supported the notion that spontaneous brain activities might serve as neuroimaging biomarkers for cognitive impairment in patients who undergone treatment of chemotherapeutic drugs. Moreover, these results provided an important basis for the developing interventions for cognitive rehabilitation of cancer patients.

## Data availability statement

The raw data supporting the conclusions of this article will be made available by the authors, without undue reservation.

## Ethics statement

This study was reviewed and approved by The Research Ethics Committee of Jiangsu Cancer Hospital & Jiangsu Institute of Cancer Research & The Affiliated Cancer Hospital of Nanjing Medical University. The patients/participants provided their written informed consent to participate in this study.

## Author contributions

SiL, ShL and XL designed the experiments. SiL, YG, JN, NY, CL, XP, RM and JW contributed to clinical data collection and assessment. SiL, YG, ShL and XL analyzed the results. SiL, YG, ShL and XL wrote the manuscript. SiL, YG, ShL and XL approved the final manuscript. All authors contributed to the article and approved the submitted version.

## Funding

The work was supported by the grants of: Jiangsu Provincial Natural Science Fund (No. BK20210977); Cadre Health Research Project of Jiangsu Province (No. BJ18033) and Foundation of Jiangsu Cancer Hospital (No. ZM201923).

## Conflict of interest

The authors declare that the research was conducted in the absence of any commercial or financial relationships that could be construed as a potential conflict of interest.

## Publisher’s note

All claims expressed in this article are solely those of the authors and do not necessarily represent those of their affiliated organizations, or those of the publisher, the editors and the reviewers. Any product that may be evaluated in this article, or claim that may be made by its manufacturer, is not guaranteed or endorsed by the publisher.
