# Supplementary material for: Chemotherapy-induced functional brain abnormality in colorectal cancer patients: a resting‐state functional magnetic resonance imaging study
Source: Front Oncol. 2022 Jul 18;12:900855. doi: 10.3389/fonc.2022.900855 (PMC9339615; doi:10.3389/fonc.2022.900855)
Supplement: Supplementary file 1 [file DataSheet_1.docx]

**1. Comparsions of fractional amplitude of low-frequency fluctuation (fALFF) values between CRC patients received different chemotherapy regimen**

Differences of fALFF values were compared between all colorectal cancer (CRC) patients including those who receiving the chemotherapy regimen of CAPOX (capecitabine plus oxaliplatin) and CAPOX plus bevacizumab and healthy controls (HCs) in this study. In addition, we also compared the differences of fALFF values between patients received different chemotherapy regimen. The participants, methods and results were presented in sTable 1.

**1.1. Participants and chemotherapy regimen**

Ten patients receiving the treatment of CAPOX and 19 patients receiving treatment of CAPOX plus bevacizumab were included to identify the differences of fALFF values between groups of patients receiving different chemotherapy regimen. Patients in the group of CAPOX were administered with CAPOX (oxaliplatin: 130 mg/m^2^ on day 1; capecitabine: 1000 mg/m^2^ twice daily on days 1 to 14; given every 3 weeks) while patients in the group of CAPOX plus bevacizumab were administered with CAPOX and bevacizumab (oxaliplatin: 130 mg/m^2^ on day 1; capecitabine: 1000 mg/m^2^ twice daily on days 1 to 14; bevacizumab: 7.5 mg/kg on day 1; given every 3 weeks). All patients received 2 to 3 months of chemotherapy with standard chemotherapeutic agents.

**1.2. Methods**

The two-sample *t*-test was conducted by the software of Resting-State fMRI Data Analysis (REST) Toolkit. The significance threshold was set at P<0.05 (voxel-level was set at P<0.001 and cluster-level was set at P<0.05; two tailed; the corresponding minimum cluster size was 8 voxels) for multiple comparisons using Gaussian Random Field (GRF) theory.

**1.3. Results**

There were no differences of fALFF values between groups of patients receiving CAPOX and CAPOX. We considered that this negative result might be related to the small sample size. Therefore, we would expand the sample size to verify this result in the future study.

**sTable 1. Demographic and clinical characteristics of patients received different chemotherapy regimen**

| **Variables** | **Patients receiving CAPOX (n=10)** | **Patients receiving CAPOX plus bevacizumab (n=19)** | ***t*/χ^2^** | ***P*** |
| --- | --- | --- | --- | --- |
| **Age (years)** | 55.20±5.59 | 59.79±9.51 | -1.40 | 0.17^a^ |
| **Gender (male/female)** | 4/6 | 10/9 | 0.42 | 0.52^b^ |
| **Education level (years)** | 14.20±1.23 | 13.95±1.75 | 0.45 | 0.65^a^ |
| **Cognitive function assessment** |  |  |  |  |
| Scores of MMSE | 25.90±1.29 | 25.47±1.71 | 0.75 | 0.46^a^ |
| Scores of MoCA | 27.50±0.97 | 26.74±0.87 | 2.08 | 0.06^a^ |
| Scores of FACT-Cog | 100.20±4.05 | 97.16±4.40 | 1.82 | 0.08^a^ |
| **Location: n (%)** |  |  |  |  |
| Colon | 4 (40%) | 1 (5%) | - | - |
| Rectum | 6 (60%) | 18 (95%) | - | - |
| **Disease stage: n (%)** |  |  |  |  |
| Ⅰ | 0 (0%) | 0 (0%) | - | - |
| Ⅱ | 2 (20%) | 1 (5%) | - | - |
| Ⅲ | 7 (70%) | 13 (69%) | - | - |
| Ⅳ | 1 (10%) | 5 (26%) | - | - |
| **Metastasis: n (%)** |  |  |  |  |
| No | 4 (40%) | 1 (5%) | - | - |
| Lung | 0 (0%) | 2 (10%) | - | - |
| Liver | 3 (30%) | 11 (59%) | - | - |
| Bone | 0 (0%) | 1 (5%) | - | - |
| Intraperitoneal | 3 (30%) | 3 (16%) | - | - |
| Pelvic | 0 (0%) | 1 (5%) | - | - |

CAPOX: capecitabine plus oxaliplatin. MMSE: Mini Mental State Exam; MoCA: Montreal Cognitive Assessment; FACT-Cog: Functional Assessment of Cancer Therapy-Cognitive Function. *P*<0.05 was considered to be statistically significant. ^a^: *P* values were obtained using two sample *t*-tests. ^b^: *P* value was obtained using the *Pearson* chi-square test.

**2. Scales of cognitive function assessment**

**2.1. MMSE**

The Mini Mental State Exam (MMSE) with 30 items was designed for screening cognitive impairment by Folstein et al. (1975). The MMSE is comprised of several short tasks. A number of areas of cognitive function can be assessed by MMSE, which includes attention, memory, language, and visuospatial abilities, as well as orientation to person, place and time.

1 point for correct answer and 0 point for wrong or unknown answer. 9 points for not suitable, 8 points for refusing to answer or not understanding. In the total score, both 8 and 9 points are calculated as 0. The maximum score is 30 points.

**2.2. MoCA**

The Montreal Cognitive Assessment (MoCA) was developed by Nasreddine based on clinical experience and with reference to the cognitive items and scores of MMSE in 2004. It is an assessment tool for rapid screening of mild cognitive impairment (MCI). MoCA includes 11 assessment items consisting of attention and concentration, executive function, memory, language, visual structure skills, abstract thinking, calculation and orientation (8 cognitive fields).

The total score is 30 points and scores ≥ 26 points indicate absence of cognitive impairment.

**2.3. FACT-Cog**

Functional Assessment of Cancer Therapy Cognitive Scale (FACT-Cog, 3rd Version) designed by Wagner et al., which is used to evaluate the self-reported cognitive function of cancer patients. FACT-Cog is comprised of 37 items, which is divided into 4 subscales consisting of 1) patients’ perceived cognitive impairments (20 items); 2) perceived cognitive abilities (9 items); 3) noticeability or comments from others (4 items); and 4) impact of cognitive changes on quality of life (4 items). The Chinese version was introduced into Chinese by Cheung et al. in 2013. Both versions of FACT-Cog (English and Chinese) have been validated within the Asian breast cancer population. The measurement equivalence of the English and Chinese versions has been confirmed, supporting the aggregation of data for analyses.

Each item is rated on a five-point Likert scale, ranging from 0 (“Never” or “Not at all”) to 4 (“Several times a day” or “Very much”). The total score for the FACT-Cog can range from 0 to 148 points, with a higher score indicative of better perceived cognitive functioning.
